# Seroprevalence and persistence of humoral immunity in response to SARS-CoV-2 vaccination and mild infection in Morocco: a cross-sectional study

**DOI:** 10.11604/pamj.2025.51.53.46802

**Published:** 2025-06-20

**Authors:** Nouhaila Najimi, Chaimae Kadi, Zainab Gaouzi, Nadia Dakka, Rabii Ameziane El hassani, Fouad Seghrouchni, Youssef Bakri

**Affiliations:** 1 Laboratory of Human Pathologies and Biology, Faculty of Sciences, Mohammed V University, Rabat, Morocco,; 2 Mohammed VI University of Sciences and Health, Casablanca, Morocco,; 3 Mohammed VI Center for Research and Innovation, Rabat, Morocco,; 4 Laboratory of Biology and Health, Faculty of Sciences of Tétouan, Abdelmalek Essaâdi University, Tétouan, Morocco,; 5 Center of Genomic Human Pathologies and Biology, Faculty of Medicine, Mohammed V University, Rabat, Morocco,; 6 Institut Supérieur des Professions Infirmières et Techniques de Santé, Rabat, Maroc

**Keywords:** Seroprevalence, humoral immunity, BBIBP-CorV vaccine, ChAdOx1 nCoV-19 vaccine, SARS-CoV-2 infection, Morocco, neutralizing antibodies, hybrid immunity, risk factors

## Abstract

**Introduction:**

assessing the seroprevalence and persistence of humoral immunity through population-based serological surveys is crucial for predicting reinfection risk and optimizing vaccination strategies. In Morocco, a mass vaccination campaign against SARS-CoV-2 has been launched to achieve herd immunity and reduce virus transmission. This study evaluated the humoral immune response to the BBIBP-CorV and ChAdOx1 nCoV-19 vaccines in healthy donors with varying doses and infection histories.

**Methods:**

IgG levels were measured via Vircell COVID-19 ELISA, and NAb levels were assessed via the GenScript Surrogate Virus Neutralization Test (sVNT). The study included individuals with different vaccination doses and prior infection histories.

**Results:**

the third vaccine dose produced the highest IgG titers, while nonvaccinated individuals and those receiving two doses had lower levels. Prior SARS-CoV-2 infection led to increased antibody levels, suggesting that hybrid immunity offers prolonged protection. The highest NAb titers were observed in individuals receiving three doses, with 96% of previously noninfected individuals being positive for NAb. Risk factor analysis indicated that prior infection (OR = 8.64, 95% CI: 2.52-29.6, p < 0.001) and three doses (OR = 22.1, 95% CI: 2.74-178, p < 0.001) increased immunity.

**Conclusion:**

hybrid immunity, involving prior infection and full vaccination, offers enhanced and longer-lasting protection. These results support the need for targeted vaccination strategies to optimize immunity and reduce the risk of adverse outcomes, especially in high-risk populations.

## Introduction

The emergence of severe acute respiratory syndrome coronavirus 2 (SARS-CoV-2) in late 2019 triggered a global pandemic of coronavirus disease 2019 (COVID-19) [1]. Vaccination remains a cornerstone of efforts to combat this crisis. In Morocco, the first vaccines introduced were ChAdOx1 nCoV-19 (AstraZeneca), an adenoviral vector-based vaccine, followed by BBIBP-CorV (Sinopharm), an inactivated virus vaccine [2]. In Morocco, research on immune responses to COVID-19 booster doses over time is still relatively limited [3]. The ChAdOx1 nCoV-19 vaccine demonstrated 76% efficacy against symptomatic COVID-19, 100% efficacy against severe disease and hospitalizations, and 85% efficacy in older adults (65+ years) after two doses [4]. Similarly, the BBIBP-CorV vaccine showed 79% efficacy against symptomatic infection and 100% efficacy against severe disease [5]. Despite these successes, studies on the long-term immune responses to booster doses of these vaccines in Morocco remain limited [6]. As of November 8, 2023, Morocco reported over 1,278,055 confirmed COVID-19 cases and 16,298 deaths [3]. With 55,389,602 vaccine doses administered [7], Morocco has achieved the highest vaccination coverage in Africa [8]. Neutralizing Antibodies (NAbs) prevent viral entry by blocking the interaction between the virus and its cellular receptor, making them essential markers of vaccine-induced protection [9,10]. While the Plaque Reduction Neutralization Test (PRNT) is the gold standard for measuring NAbs, other tests, such as the GenScript cPass™ SARS-CoV-2 Surrogate Virus Neutralization Test (sVNT), offer simpler alternatives with high specificity (99.2%) and sensitivity (80.3%). Similarly, Vircell COVID-19 ELISA IgG is effective for detecting anti-SARS-CoV-2 IgG antibodies, with a specificity of 90.2% and sensitivity of 85.7% [11,12].

Our study is one of the few in Morocco investigating the kinetics of neutralizing antibodies following three vaccine doses. We evaluated healthy donors at different post-booster times and compared the responses of previously infected and uninfected individuals. These findings provide critical insights into population-level immunity and the persistence of humoral responses over time. Morocco launched its national vaccination campaign on January 28, 2021, based on short-term randomized controlled trials demonstrating vaccine efficacy rates of 62%-96% against symptomatic disease after two doses [13,14]. However, real-world studies are essential to complement trial data. Serological studies are valuable tools for assessing vaccination campaigns and monitoring immune responses [15,16]. These studies are particularly important in Morocco, where factors such as genetic diversity, due to a high rate of consanguineous marriages, contribute to a unique genetic landscape [17]. Comorbidities such as familial Mediterranean fever, cystic fibrosis, and beta-thalassemia, along with logistical challenges such as limited access to advanced genetic diagnostics, may also influence immune responses [18]. Additionally, understanding the duration of immunity is crucial for assessing long-term vaccine effectiveness.

Given the heterogeneous nature of Morocco's population, our study focused on healthy donors to establish a baseline of population-level immunity. Factors such as genetic diversity and comorbidities require specific, targeted studies to fully understand their impact. This study contributes to the limited body of literature on the long-term immunogenicity of booster doses in Morocco. These findings underscore the importance of booster timing and targeted vaccination strategies for optimizing public health interventions.

## Methods

**Study design:** this study was designed as a cross-sectional seroprevalence assessment to evaluate humoral immunity in response to SARS-CoV-2 vaccination and mild infection in Morocco. This study aimed to determine the levels of SARS-CoV-2-specific antibodies in individuals with varying vaccination histories and prior infections.

**Study setting and population:** serum samples were separated from blood collected from 294 participants to measure SARS-CoV-2 antibodies. Thirty-three participants were excluded due to severe COVID-19 infection, leaving 261 healthy donors. These donors voluntarily donated blood at the Transfusion Blood Center of Rabat between April 2021 and March 2023. The study included individuals from regions around Rabat-Salé, as well as other Moroccan cities. The group included individuals with diverse profiles based on vaccination status and prior infection: nonvaccinated controls; individuals vaccinated with one or more doses of the SARS-CoV-2 BBIBP-CorV vaccine or the ChAdOx1 nCoV-19 vector vaccine; those with a history of prior SARS-CoV-2 infection; and individuals with heterogeneous vaccine types.

**Variables:** the independent variables in this study included vaccination status, prior SARS-CoV-2 infection, and demographic characteristics. The dependent variables were IgG antibody titers, which were measured via Vircell COVID-19 ELISA, and Neutralizing Antibodies (NAbs), which were assessed via the GenScript Surrogate Virus Neutralization Test (sVNT). To ensure the robustness of our findings, we considered potential confounders, such as age and sex, and accounted for them in our analysis.

### Data resources and measurement

**Data collection tool:** a structured questionnaire was used to collect sociodemographic data, clinical and medical history, potential risk factors for SARS-CoV-2 infection, and details about participants' health history. The questionnaire included self-reported information on prior COVID-19 infection (confirmed by PCR), vaccination status, and comorbidities.

**Data collection:** blood samples were collected from participants after providing written informed consent. The serum was separated and stored for antibody testing. To maintain confidentiality, personal identifiers were removed and replaced with coded identifiers. The questionnaire responses and laboratory test results were recorded systematically for data analysis.

**Subject exclusion criteria:** subjects with a confirmed history of severe COVID-19 infection, including hospitalization and associated complications. Individuals with a history of chronic or acute illnesses could confound the study results. Participants who did not provide informed consent or withdrew consent during the study. Subjects with incomplete or unreliable data records.

**Subject inclusion criteria:** participants aged 18 years and above were recruited if they were mild, asymptomatic, or had no history of SARS-CoV-2 infection. Individuals who provided informed consent to participate in the study. Subjects with a confirmed history of mild COVID-19 infection based on self-reported symptoms or clinical diagnosis. Participants with no history of COVID-19 infection, confirmed by the absence of clinical symptoms and/or serological evidence. Asymptomatic individuals were confirmed to be negative for SARS-CoV-2 infection through RT-PCR or serological testing at the time of recruitment.

### Serological tests

**Enzyme-Linked Immunosorbent Assay (ELISA):** the Receptor-Binding Domain (RBD) of the SARS-CoV-2 spike protein is a key immunodominant region, indicating that immune responses against the RBD are reliable markers of vaccine-induced immunity. Our test also detects Abs against the Nucleocapsid Protein (NP), which enables the identification of immune responses resulting from natural infection [19]. To assess IgG antibody levels, serum samples (n=261) were analyzed at the Biology of Human Pathologies Laboratory, Faculty of Science, Rabat, Morocco, via the Vircell COVID-19 ELISA IgG Kit (Vircell Spain S.L.U., Granada, Spain). The assay measured antibody reactivity to both the spike-RBD and the NP antigens, providing a comprehensive overview of immune responses. The absorbance was measured at 450/620 nm, and the results were categorized as negative (<4), ambiguous (4-6), or positive (>6) based on the manufacturer´s guidelines.

**Neutralization assay:** the surrogate virus neutralization test (sVNT) (GenScript cPass™ SARS-CoV-2 Neutralization Antibody Detection Kit). The Netherlands was used to detect neutralizing antibodies that block the interaction between the SARS-CoV-2 spike RBD and the ACE2 receptor. The assay was performed per the manufacturer´s recommendations on 176 selected samples. The absorbance was measured at 450 nm. The results were interpreted according to the manufacturer´s criteria: NAbs were considered present if the inhibition signal exceeded 30%.

**Sample size:** a total of 294 participants were initially recruited, with 33 excluded due to severe infection, resulting in a final study population of 261 healthy donors. The sample size was determined through power calculations to ensure adequate representation of the vaccination and infection profiles. Donor availability is limited by socioeconomic factors, making recruitment challenging, which impacts the diversity of the donor pool.

**Statistical analysis:** demographic data were analyzed via R software with the appropriate packages. Univariate and multivariate logistic regression analyses were conducted in R to estimate Odds Ratios (ORs), with IgG antibody status as the outcome variable. Statistical analyses were performed via Prism 8.0.1 (GraphPad Software, USA). The Mann-Whitney U test was used to assess the significance between groups, whereas the Spearman correlation coefficient was used to evaluate the association between Vircell COVID-19 ELISA IgG and sVNT levels. A p-value of < 0.05 was considered statistically significant. Quantitative variables were analyzed via statistical methods to compare groups and assess correlations. IgG titers and NAb levels were treated as continuous variables. Age, vaccine dose, and time since infection/vaccination were examined as continuous and categorical variables in regression models.

**Ethical consideration:** for this study, it was obtained from Biomedical Research (CERB) of the Faculty of Medicine and Pharmacy in Rabat, BOARD (APPROVAL NUMBER/ID): 28/20.

## Results

**Demographic data:** the study included 261 participants with a mean age of 46.5 years (SD = 13.7) and a median age of 47.0 years, ranging from 19-95 years. Among them, 42.1% were female, and 57.5% were male. The age distribution revealed that 13.0% were aged 18-29 years, 43.3% were aged 30-49 years, 33.7% were aged 50-64 years, and 8.4% were aged 65 years or older (Table 1). Additional demographic data are presented in Table 2.

**Table 1:** overall demographic characteristics of the participants

	Overall (N=261)
**Age (years)**	
Mean (SD)	46.5 (13.7)
Median [Min, Max]	47.0 [19.0, 95.0]
Missing	4 (1.5%)
**Sex**	
F	110 (42.1%)
M	150 (57.5%)
Missing	1 (0.4%)
**Age groups years**	
18-29	34 (13.0%)
30-49	113 (43.3%)
50-64	88 (33.7%)
65	22 (8.4%)
Missing	4 (1.5%)

**Table 2:** distribution of participants based on positive and negative seroprevalence outcomes

	Anti-Spike RBD Anti-NP negative (N=44)	Anti-Spike RBD Anti-NP positive (N=193)	Overall (N=237)	p value
**Age**				
Mean (SD)	42.7 (13.6)	47.8 (13.8)	46.8 (13.9)	0.0121
Median [Min, Max]	44.0 [22.0, 95.0]	48.0 [19.0, 87.0]	48.0 [19.0, 95.0]	
Missing	0 (0%)	4 (2.1%)	4 (1.7%)	
**Gender**				0.0702
F	14 (31.8%)	90 (46.6%)	104 (43.9%)	
M	30 (68.2%)	102 (52.8%)	132 (55.7%)	
Missing	0 (0%)	1 (0.5%)	1 (0.4%)	
**Age groups (years)**				0.1143
18-29	9 (20.5%)	21 (10.9%)	30 (12.7%)	
30-49	21 (47.7%)	81 (42.0%)	102 (43.0%)	
50-64	13 (29.5%)	66 (34.2%)	79 (33.3%)	
≥65	1 (2.3%)	21 (10.9%)	22 (9.3%)	
Missing	0 (0%)	4 (2.1%)	4 (1.7%)	
**Infection state**				< .0012
Previously infected	3 (6.8%)	67 (34.7%)	70 (29.5%)	
Not previously infected	29 (65.9%)	75 (38.9%)	104 (43.9%)	
Missing	12 (27.3%)	51 (26.4%)	63 (26.6%)	
**Vaccine type**				< .0013
ChAdOx1 nCoV-19 (AstraZeneca)	21 (47.7%)	67 (34.7%)	88 (37.1%)	
BBIBP-CorV (Sinopharm)	9 (20.5%)	73 (37.8%)	82 (34.6%)	
Not Vaccinated	14 (31.8%)	26 (13.5%)	40 (16.9%)	
Heterogenous	0 (0%)	24 (12.4%)	24 (10.1%)	
Missing	0 (0%)	3 (1.6%)	3 (1.3%)	
**Booster doses**				< .0013
No dose	14 (31.8%)	26 (13.5%)	40 (16.9%)	
One dose	0 (0%)	9 (4.7%)	9 (3.8%)	
Two doses	29 (65.9%)	109 (56.5%)	138 (58.2%)	
Three doses	1 (2.3%)	41 (21.2%)	42 (17.7%)	
Four doses	0 (0%)	2 (1.0%)	2 (0.8%)	
Missing	0 (0%)	6 (3.1%)	6 (2.5%)	
**Post-booster time**				0.0303
Less than 14 days post-vaccination	2 (4.5%)	13 (6.7%)	15 (6.3%)	
14 to 31 days postvaccination	14 (31.8%)	31 (16.1%)	45 (19.0%)	
31 days postvaccination	14 (31.8%)	70 (36.3%)	84 (35.4%)	
More than 8 months after vaccination	1 (2.3%)	25 (13.0%)	26 (11.0%)	
Missing	13 (29.5%)	54 (28.0%)	67 (28.3%)	

1 Wilcoxon, 2 Chi-square and 3 Fisher

**Figure 1:**
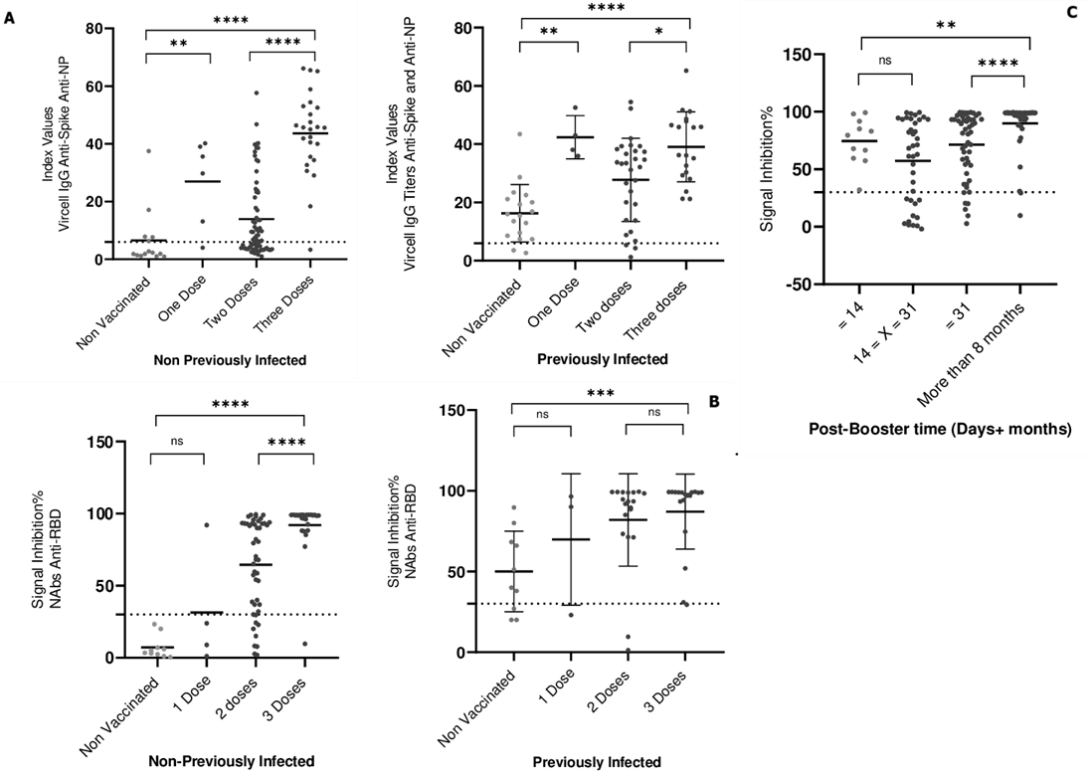
humoral response kinetics to two vaccine platforms; the inactivated SARS-CoV-2 BBIBP-CorV vaccine and the ChAdOx1 nCoV-19 vector vaccine in PNI individuals and previously infected individuals: A) anti-Spike RBD and Anti-NP IgG Antibody Response; B) neutralizing antibody levels to two vaccine platforms, the inactivated SARS-CoV-2 BBIBP-CorV vaccine and the ChAdOx1 nCoV-19 vector vaccine, in previously infected and previously noninfected patients; C) seroneutralizing protection against the RBD at different time points. ns = not significant; * = p < 0.05; ** = p < 0.01; *** = p < 0.001; **** p=0.0001; the positivity threshold is represented by a dotted line

**Seroprevalence and kinetics of anti-SARS-CoV-2 IgG and neutralizing antibodies across vaccination profiles:** we assessed the seroprevalence and kinetics of anti-SARS-CoV-2 IgG (spike and NP) and NAbs in 237 participants across different vaccination profiles (unvaccinated, one dose, two doses, and three doses of BBIBP-CorV or ChAdOx1 nCoV-19) and prior infection status. Table 2 shows seroprevalence data, with 44 negative and 193 positive individuals. The positive group had a mean age of 47.8 years (vs. 42.7 years in the negative group, p = 0.0121). Prior infection was more common in the positive group (34.7% vs. 6.8%, p < 0.0012), and the proportion of Sinopharm recipients was greater. Additionally, booster timing influenced the results, with 13% of individuals who boosted >8 months ago testing positive (p = 0.0303). Among uninfected individuals, third-dose recipients had significantly higher median IgG titers (29.74) than did those in the one-dose (2.28) and two-dose groups (p < 0.01 and p = 0.0001). A similar trend was observed in previously infected individuals, with third-dose recipients showing higher IgG titers (p = 0.0001). Interestingly, one-dose recipients had slightly higher median IgG titers than third-dose recipients did (36.21 vs. 34.66, p < 0.05) (Figure 1 A).

**Neutralizing antibodies:** ninety-six percent of the third-dose recipients had detectable NAbs (median 98.63), whereas 80.85% had detectable NAbs in the two-dose group (median 90%, p = 0.0001), and 25% had detectable NAbs in the one-dose group. Nonvaccinated individuals presented negligible NAb (median 4.2). Among previously infected individuals, NAb levels increased with increasing vaccine dose (medians: 50.01 for one dose, 79.91 for two doses, 92.59 for three doses, and 97.58 for the boosted group) (Figure 1 B). Booster timing also influenced NAb levels, with the highest concentrations observed more than 8 months postvaccination, followed by 14 to 31 days postbooster. The durability of immunity beyond 31 days remains unclear and warrants further investigation (Figure 1 C).

**Figure 2:**
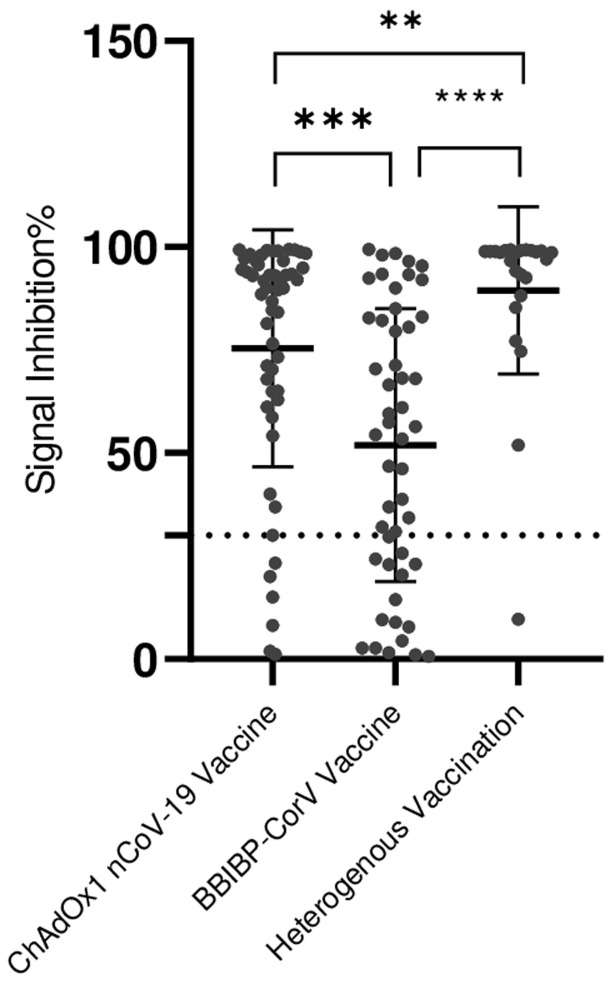
vaccine protection; BBIBP-CorV and ChAdOx1 nCoV-19 vaccine platforms are the most commonly used in Morocco: ns = not significant; *= p < 0.05; **= p < 0.01; ***= p < 0.001; ****p=0.0001 (The positivity threshold is represented by a dotted line)

**Vaccine type and immune protection:** the median NAb seroconversion level in vaccinated donors was 67.35 for those who received the BBIBP-CorV vaccine and 62.09 for those vaccinated with ChAdOx1 nCoV-19 (p < 0.001). A positivity rate of 73.52% was observed in donors who received the BBIBP-CorV vaccine, whereas 90% positivity was detected in those who received the ChAdOx1 nCoV-19 vaccine. Additionally, higher levels of neutralizing antibodies were detected in donors with heterogeneous vaccination, with a median of 98.57 (Figure 2).

**Figure 3:**
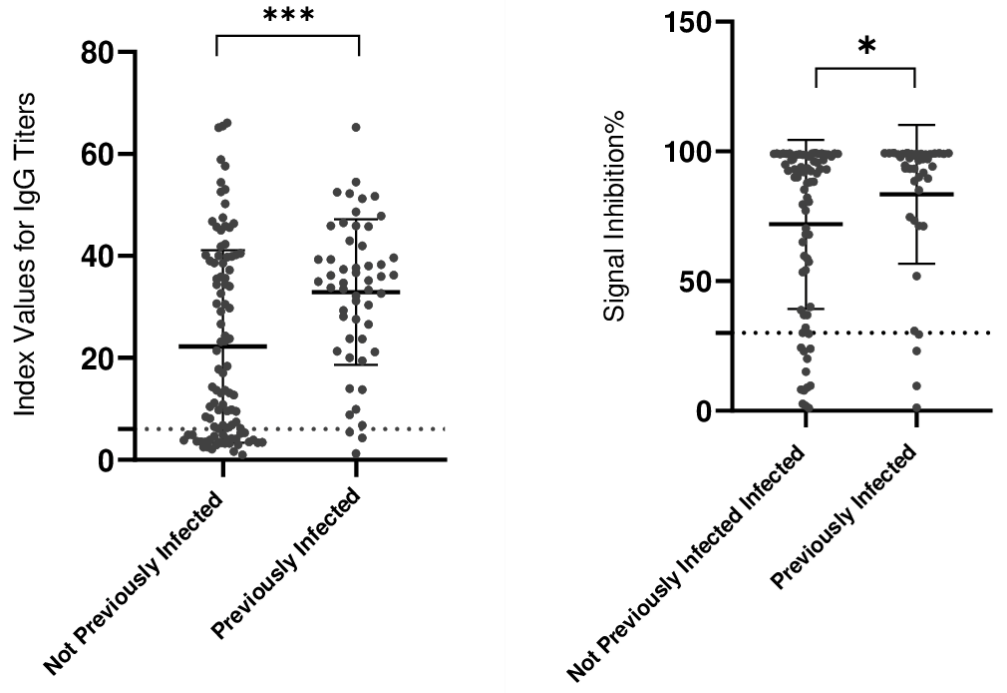
NAb levels against RBD and IgG anti-Spike and anti-NP Abs in hybrid immunity donors: ns = not significant; *= p < 0.05; **= p< 0.01; ***= p < 0.001; ****p=0.0001 (The positivity threshold is represented by a dotted line)

**Hybrid immunity:** IgG responses against spike, nucleocapsid, and NAb in vaccinated/infected donors: seropositivity measurements revealed that hybrid immunity donors had a significantly greater median value (34.9) than previously noninfected individuals did (13.6) (p < 0.001). This difference was less pronounced but still significant when NAb was evaluated, with hybrid immunity donors showing a higher median (83.4) than previously noninfected individuals (71.8) (p < 0.05) (Figure 3).

**Figure 4:**
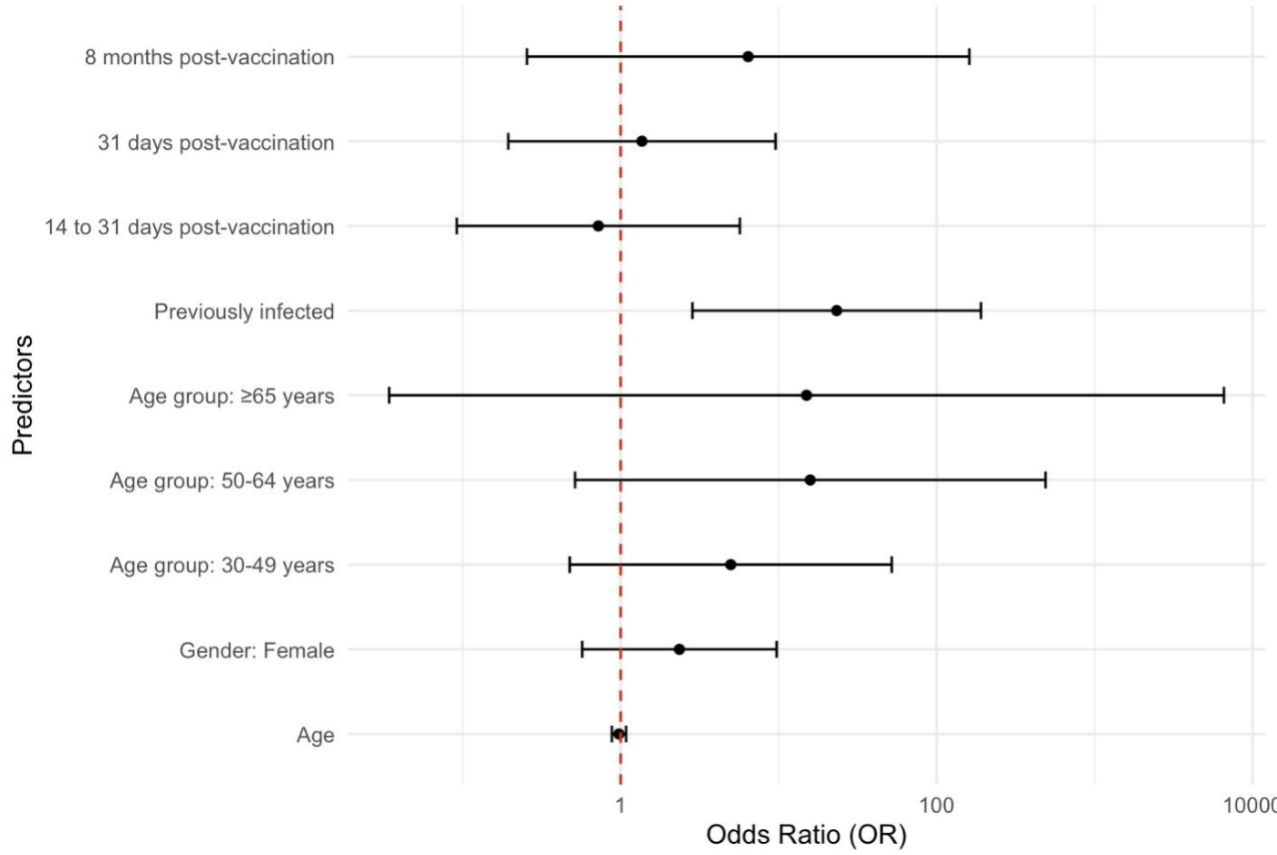
odds ratio plots from logistic regression predicting outcomes for the multivariate analysis of factors influencing seroprevalence (Dots represent the odds ratios derived from the regression analysis in table 2, and lines represent 95% confidence intervals)

**Factors influencing seroprevalence outcomes:** according to the univariate analysis, age (≥65) was significantly associated with seropositivity for anti-SARS-CoV-2 antibodies (OR = 9.00, 95% CI: 1.05-77.5, p = 0.0454). Gender was not statistically significant, but females had greater odds (OR = 1.89, 95% CI: 0.944-3.79, p = 0.0724). Previous infection was a strong predictor (OR = 8.64, 95% CI: 2.52-29.6; p < 0.001). Compared with the ChAdOx1 nCoV-19 vaccine (AstraZeneca), the BBIBP-CorV (Sinopharm) vaccine had greater odds (OR = 4.37, 95% CI: 1.69-11.3, p = 0.00234). Booster doses increased odds, with three strongly associated doses (OR = 22.1, 95% CI: 2.74-178, p = 0.00316), and postvaccination timing was associated with increased odds for those >8 months postbooster (OR = 11.3, 95% CI: 1.39-91.8, p = 0.0234) (Table 3, Figure 4). In the multivariate analysis, after adjusting for other factors, age (≥65) and sex were no longer significant. Previous infection remained a strong predictor (OR = 23.3, 95% CI: 2.85-191, p = 0.00334). The 14- to 31-day postvaccination period was associated with reduced odds of seropositivity (OR = 0.758, 95% CI: 0.723-0.920, p < 0.05), but other postvaccination intervals showed no significant effects (Table 3, Figure 4).

**Table 3:** univariate and multivariate analyses for the association between risk factors and SARS-CoV-2 seropositivity from the transfusion blood center of Rabat from April 2021 to March 2023

Variables	Univariate Analysis		Multivariate Analysis	
	OR (95%)	p value	OR (95%)	p value
**Age groups (years)**	1.03 (1.00-1.06)	0.0281	0.977 (0.880- 1.08)	
18-29	1.00		1.00	
30-49	1.65 (0.661-4.13)	0.282	4.98 (0.477-52.0)	0.180
50-64	2.18 (0.815-5.81)	0.121	15.9(0.515 -489.0)	0.114
≥65	9.00 (1.05-77.5)	0.0454	15.0 (0.0343-6582)	0.383
**Gender**				
Male	0.529 (0.264-1.06)	0.0724	1.00	
Female	1.89 (0.944- 3.79)	0.0724	2.36 (0.571-9.72)	0.236
**Previously infected**				
No	0.116 (0.0337- 0.398)	< .001	1.00	
Yes	8.64 (2.52-29.6)	< .001	23.3 (2.85-191)	0.00334
**Vaccine type**				
ChAdOx1 nCoV–19 (AstraZeneca)	1.72 (0.761-3.88)	0.193	-	-
BBIBP–CorV (Sinopharm)	4.37 (1.69-11.3)	0.00234	-	-
**Booster doses**				
One dose	-	-	-	-
Two Doses	2.02 (0.939- 4.36)	0.00367	-	-
Three Doses	22.1 (2.74- 178.)	0.00316	-	
Four Doses	-	-	-	-
**Post-booster time**				
Less than 14 days post-vaccination	2.94 (0.583-14.8)	0.192	1.00	
14 to 31 days postvaccination	1.00		0.758 (0.723-0.0920)	-
31 days post-first vaccination	2.26 (0.962 -5.30)	0.0613	0.754 (1.37- 0.195)	0.314
More than 8 months after vaccination	11.3 (1.39- 91.8)	0.0234	6.42 (0.256 -161.0)	0.258

## Discussion

Morocco's mass vaccination campaign against SARS-CoV-2 aims to increase herd immunity and reduce transmission. The approved vaccines have demonstrated strong safety and efficacy, significantly lowering hospitalizations and deaths [13,20]. Understanding the humoral immune response postvaccination remains critical for predicting reinfection risk and enhancing pandemic control strategies [8]. This knowledge enables public health officials to design more effective vaccination programs tailored to population-specific needs, thereby ensuring sustained immunity and preparedness for emerging variants. Recent modeling studies emphasize the importance of incorporating immunological data into vaccination strategy optimization, especially for booster campaigns and prioritizing high-risk populations [21,22]. Evidence from multicountry studies also shows that aligning vaccination schedules with seroprevalence and neutralizing antibody persistence can maximize impact and reduce health disparities [23,24].

**IgG responses and neutralizing antibodies across vaccine doses and infection profiles:** among Previously Noninfected Individuals (PNI), the highest IgG titers were observed in those who received a third vaccine dose, followed by the first dose in seropositive donors. The lowest levels were observed in nonvaccinated individuals and those receiving two doses. These findings are consistent with other studies on BBIBP-CorV vaccine recipients [25]. For Previously Infected (PI) individuals, higher antibody levels were observed, suggesting that vaccination following natural infection provides more robust and prolonged protection [26]. In both the PI and PNI groups, three doses led to similar high IgG titers. We demonstrated the presence of neutralizing antibodies via the GenScript SARS-CoV-2 surrogate virus neutralization test, which demonstrated high sensitivity, specificity, and strong correlation with the gold standard [27]. The highest NAb titers were observed in PNI individuals who received three doses, with 96% testing positive for neutralizing antibodies. A similar trend was observed in PI donors, where those who received three doses had higher median NAb titers than those with two doses or no vaccine. This finding reinforces the positive impact of the vaccination regimen in enhancing the immune response, particularly following the third dose. This finding is consistent with studies showing higher titers in individuals who received a third dose than in those who received two doses [28-30]. This study confirmed that not only are IgG antibodies present for the two viral antigens but also that a significant proportion of these antibodies can neutralize the virus, thereby protecting the host.

**Neutralizing antibody trends and longevity:** this study tracked neutralizing antibody levels at four time points postvaccination and revealed that the median levels remained relatively stable overall. Despite this, individual responses varied, with the highest NAb concentrations observed in some individuals more than 8 months after vaccination. This suggests that NAb responses can persist or even increase over time and that booster doses may not always elicit immediate surges. These results align with findings in previously infected individuals showing stronger and longer-lasting IgG responses than infection-naïve individuals did [31]. Collectively, these data support the optimization of vaccine schedules and the prioritization of third-dose boosters, especially in uninfected populations. The incorporation of serological insights into rollout strategies could improve the equity and effectiveness of public health interventions [21-24]. The findings of this study provide essential data that can help shape vaccination policy in Morocco and beyond. For example, data suggesting higher antibody responses after the third dose, particularly among PNI individuals, can inform the optimization of booster campaigns, ensuring that high-risk populations, such as elderly individuals or those with chronic conditions, receive the necessary protection. Additionally, given the persistent nature of NAb responses in some individuals, the strategy for vaccine boosting could be adjusted to focus on those with lower immunity, balancing the need for timely administration of boosters [32,33].

**Hybrid immunity and B- and T-cell responses:** humoral immunity is not the only protective response against infection. Patients who received the CoronaVac vaccine and produced NAbs were found to have much higher levels of anti-spike IgG and a propensity to produce more spike-specific memory B cells than nonresponders did [34]. Furthermore, following the BBIBP-CorV vaccine, seropositive individuals also express long-lasting T-cell memory, such as CD8+ effector T, anti-S, anti-NP, and membrane proteins of the virus [35]. A total of 51.17% of the study population had a history of both prior SARS-CoV-2 infection and subsequent vaccination. This means that a significant portion of the population had experienced both natural infection and vaccine-induced immunity. We noted that individuals with hybrid immunity have a higher median seropositivity than those who were previously noninfected. These findings correlate with a study that concluded that people who were previously exposed to SARS-CoV-2 respond to vaccination with more B-cell-producing antibodies that are not susceptible to escape variants and have greater neutralization potency [36]. We also found a statistically significant difference in Nab levels between individuals who had hybrid immunity and those who were previously noninfected. However, the difference in NAb levels was smaller than the significant disparity in seropositivity rates between the two groups. This suggests that while hybrid immunity is generally linked to higher NAb levels, the effect on IgG antibodies is less pronounced. Hybrid immunity, resulting from both natural infection and vaccination, provides a more robust and long-lasting immune response [37]. This finding has implications for future vaccination strategies, particularly in populations with prior exposure to the virus. It could be argued that individuals with hybrid immunity might require fewer booster doses or may benefit from different types of vaccines [38], which could help optimize resource allocation and reduce vaccine waste.

**Vaccine platform comparison:** in the present study, there was a significant difference in neutralizing antibody levels between individuals vaccinated with the BBIBP-CorV vaccine and those vaccinated with the ChAdOx1 nCoV-19 vaccine. The NAb levels for BBIBP-CorV appear to be higher than those for ChAdOx1 nCoV-19. The proportion of positive donors who received BBIBP-CorV was 73.52%. For those who received ChAdOx1 nCoV-19, the proportion of positive samples was greater at 90%. The same result was reported in a study where the seroconversion rate for anti-Spike RBD antibodies was significantly greater in the ChAdOx1 nCoV-1 vaccine group than in the BBIBP-CorV vaccine group. There are even higher levels of neutralizing antibodies in donors who receive heterogeneous vaccinations. However, there was no significant correlation between the serological responses to the two vaccines. This means that there is no strong linear relationship between the serological responses induced by the BBIBP-CorV and ChAdOx1 nCoV-19 vaccines. This finding was also reported in a study comparing the two vaccine platforms. They noted that the total antibody level did not significantly differ between the two groups [39]. The observed difference in neutralizing antibody levels between BBIBP-CorV and ChAdOx1 nCoV-19 highlights the need to tailor vaccination strategies. While BBIBP-CorV elicited stronger responses after multiple doses, the link between vaccine type and long-term immunity remains complex, warranting further investigation to guide platform selection for different populations.

**Seroprevalence risk factors:** this study revealed significant associations between seroprevalence and age, prior infection, vaccine type, booster dose, and postbooster timing. Individuals aged 65 and older had a higher odds ratio, indicating a greater risk of adverse outcomes, which is consistent with findings that older adults are more vulnerable to severe COVID-19 [40]. Older individuals have been consistently identified as a high-risk group for severe disease and poor outcomes, as immunity tends to weaken with age [40]. Future vaccination campaigns could prioritize booster doses for individuals aged 65 and older [41]. Although sex did not reach statistical significance, there was a greater likelihood of adverse outcomes in females. This result aligns with a study conducted in Morocco, in which women presented higher antibody levels than men did [8]. Some studies have suggested increased susceptibility to COVID-19 in males, often referred to as “male bias,” which may be partly explained by changes in sex steroid concentrations and immune system aging [42]. Prior infection emerged as a strong predictor, with previously infected individuals showing much higher odds, supporting the role of natural immunity in reducing reinfection severity [43]. Vaccine type analysis revealed increased odds for BBIBP-CorV (Sinopharm) recipients, likely due to different immune responses elicited by various vaccines [44]. A clear association was observed with booster doses: three doses were linked to reduced adverse outcomes [45]. The time since the last booster also plays a crucial role. Individuals over 8 months postvaccination had significantly increased odds, indicating waning immunity over time [46]. This finding emphasizes the importance of considering booster intervals to maintain immunity levels, especially in high-risk groups. Conversely, in the early postvaccination interval of 14-31 days, individuals displayed reduced odds, suggesting a period of enhanced immunity shortly after receiving the booster dose. After adjusting for all factors, certain associations observed in the univariate analysis became less pronounced. For example, the significance of age diminished, and the OR for individuals aged 65 and above was no longer statistically significant. Similarly, gender remained nonsignificant, indicating that when controlling for other variables, age and gender may have a limited impact on outcomes. However, prior infection continues to be a strong predictor, reinforcing the role of natural immunity in reducing risk [47].

**Limitations:** our study has several limitations. First, the participants were all healthy donors from a blood transfusion center, mostly without comorbidities, meaning that the findings may not be generalizable to the broader population. Second, we focused solely on the wild-type SARS-CoV-2 virus and did not assess vaccine efficacy against emerging variants. Furthermore, we did not assess cellular T and B memory responses in coordination with humoral immunity, limiting our understanding of the complete immune response. Finally, the number of individuals who received only one dose was small because of the implementation of a two-dose vaccination policy in Morocco during the first recruitment, reducing the number of participants in this group.

## Conclusion

This study is one of the few conducted in Morocco on a healthy donor population, particularly those receiving three doses of COVID-19 vaccines, and it tracks the dynamics of humoral responses over time. These findings highlight the importance of booster doses and suggest that booster timing is crucial for maintaining optimal immunity levels. Our results align with those of global studies showing that natural immunity combined with vaccination provides the most robust protection against reinfection and severe disease. These insights are essential for informing public health strategies and vaccine planning.

### What is known about this topic

- Compared with vaccination alone, hybrid immunity (natural infection + vaccination) provides stronger and longer-lasting protection against SARS-CoV-2;
- The third vaccine dose significantly enhances neutralizing antibody titers, particularly in previously noninfected individuals;
- Different vaccine platforms induce varying humoral responses, with heterogeneous vaccination regimens resulting in increased antibody responses.

### What this study adds

- This study provides quantitative evidence that a third vaccine dose significantly enhances both IgG and neutralizing antibody responses, with the most substantial increases observed in individuals without prior SARS-CoV-2 infection;
- It confirms the immunological advantage of hybrid immunity, while offering novel insights into how booster timing and infection history influence the magnitude of the humoral response; it confirms the immunological advantage of hybrid immunity, while offering novel insights into how booster timing and infection history influence the magnitude of the humoral response;
- Importantly, the analysis indicates that age and sex have a minimal impact on antibody levels when infection history and vaccine regimen are taken into account an aspect not extensively addressed in prior literature.
